# Effects of remote limb ischemic conditioning on muscle strength in healthy young adults: A randomized controlled trial

**DOI:** 10.1371/journal.pone.0227263

**Published:** 2020-02-04

**Authors:** Swati M. Surkar, Marghuretta D. Bland, Anna E. Mattlage, Ling Chen, Jeffrey M. Gidday, Jin-Moo Lee, Tamara Hershey, Catherine E. Lang

**Affiliations:** 1 Program in Physical Therapy, Washington University School of Medicine, St. Louis, MO, United States of America; 2 Division of Biostatistics, Washington University School of Medicine, St. Louis, MO, United States of America; 3 Departments of Ophthalmology, Physiology, and Neuroscience, Louisiana State University Health Sciences Center, New Orleans, LA, United States of America; 4 Department of Neurology, Washington University School of Medicine, St. Louis, MO, United States of America; 5 Department of Psychiatry, Washington University School of Medicine, St. Louis, MO, United States of America; 6 Program in Occupational Therapy, Washington University School of Medicine, St. Louis, MO, United States of America; Fondazione Toscana Gabriele Monasterio, ITALY

## Abstract

Remote limb ischemic conditioning (RLIC) is a clinically feasible method in which brief, sub-lethal bouts of ischemia protects remote organs or tissues from subsequent ischemic injury. A single session of RLIC can improve exercise performance and increase muscle activation. The purpose of this study, therefore, was to assess the effects of a brief, two-week protocol of repeated RLIC combined with strength training on strength gain and neural adaptation in healthy young adults. Participants age 18–40 years were randomized to receive either RLIC plus strength training (n = 15) or sham conditioning plus strength training (n = 15). Participants received RLIC or sham conditioning over 8 visits using a blood pressure cuff on the dominant arm with 5 cycles of 5 minutes each alternating inflation and deflation. Visits 3–8 paired conditioning with wrist extensors strength training on the non-dominant (non-conditioned) arm using standard guidelines. Changes in one repetition maximum (1 RM) and electromyography (EMG) amplitude were compared between groups. Both groups were trained at a similar workload. While both groups gained strength over time (P = 0.001), the RLIC group had greater strength gains (9.38 ± 1.01 lbs) than the sham group (6.3 ± 1.08 lbs, P = 0.035). There was not a significant group x time interaction in EMG amplitude (P = 0.231). The RLIC group had larger percent changes in 1 RM (43.8% vs. 26.1%, P = 0.003) and EMG amplitudes (31.0% vs. 8.6%, P = 0.023) compared to sham conditioning. RLIC holds promise for enhancing muscle strength in healthy young and older adults, as well as clinical populations that could benefit from strength training.

## Introduction

Ischemic conditioning describes a phenomena in which an organ exposed to a controlled, short-term, local, sublethal ischemia will be protected from subsequent ischemia [1–4]. Remote ischemic conditioning is another more practical approach where transient ischemia and reperfusion applied to a remote organ or tissue, protects other organs or tissues from further episodes of lethal ischemia/reperfusion injury. For example, ischemia on the limb protects the brain from subsequent ischemic injury [5–7]. Ischemic conditioning can be delivered before (preconditioning), during (perconditioning) or after (postconditioning) the ischemic insult [8]. Remote limb ischemic conditioning (RLIC) is a clinically feasible way of performing remote ischemic conditioning where alternating, brief ischemia and reperfusion is delivered with cyclic inflation and deflation of a blood pressure cuff on the arm or leg [9].

In 1986, ischemic conditioning was first found to protect cardiac myocytes from ischemia [10–12]. Since then a number of studies have found that ischemic conditioning also protects skeletal muscles from infarction in porcine and rodent models [13, 14]. Subsequent studies in humans have shown that RLIC improves exercise performance in healthy young adults [15–18]. Specifically, a single session of repeated 5-min bouts of RLIC, not combined with any training, increases muscle force [19], power output [20], and muscular endurance [21, 22], delays muscle fatigue [23], and improves recovery time between tasks that require maximum force generation [24]. In stroke survivors, also within a single session, RLIC has shown to increase paretic muscle force and activation [25]. In a similar paradigm delivered over two weeks, RLIC improved walking capacity and decreased neuromuscular fatigability, despite failing to increase muscle force [26].

Although the specific mechanisms for improving skeletal muscle performance are incompletely understood, the beneficial effects of RLIC on skeletal muscles occurring secondary to humoral, neural and metabolic mechanisms are generally accepted [27]. Specifically, RLIC improves metabolic efficiency by reducing adenosine triphosphate (ATP) and glycogen depletion [13, 14, 28–30], increasing blood flow by inducing vasodilation [20, 31, 32], and decreasing lactate production [15, 18]. RLIC also lowers sensitivity to fatigue signals [17], and increases neural drive, and the recruitment of motor units [33, 34].

These studies open the possibility that RLIC, when paired with skeletal muscle training, could enhance strength gains in humans. Improving muscle strength is important for many populations such as those undergoing rehabilitation [35–37], prolonged inactivity [38, 39], chronic diseases [40, 41], and aging [42]. In short, if RLIC can be harnessed to enhance strength training, many people around the world would benefit. Considering the beneficial effects of RLIC alone on skeletal muscle, pairing RLIC with strength training merits further investigation.

The purpose of this study, therefore, was to determine whether RLIC could enhance a brief period of muscle strength training in healthy young adults. We note that paring RLIC with strength training is a different technique than the blood flow restriction (BFR) exercise paradigms popularized by high performance athletes [43–47]. The latter are characterized by limiting venous blood flow to the working muscle, but do not generate ischemic bouts in the muscle [48–50]. BFR exercise paradigms target the muscle that receives the flow restriction, whereas RLIC operates systemically [22, 48]. A short-duration, high-intensity strength training protocol was selected in this early phase study and thus is not intended as a comprehensive evaluation. We chose to test healthy young adults to determine if benefits occur with healthy physiology before moving to specific, co-morbid patient populations. We hypothesized that combining RLIC with strength training would enhance strength gains compared to sham conditioning. Results from this study can be used to inform future, more definitive studies of this combinatorial therapy.

## Materials and methods

### Trial design

This study was an early phase, prospective, single-blind, randomized controlled trial with a repeated-measures design. The study was approved by Washington University Human Research Protection Office on 09/12/2017 and conducted from 11/08/2017 to 12/18/2018. The trial was registered at https://www.clinicaltrials.gov/ (NCT03512028). The definition of a clinical trial and the rules for registration changed after the study was initiated. At the time of design, funding, and initiation, the study was considered as an experiment. Due to the changes in the definition of clinical trials at NIH, we have registered the study as clinical trial after enrolling few participants. All related trials for this intervention are registered at clinicaltrials.gov.

The study included ten total visits to assess the combined effects of remote limb ischemic conditioning (RLIC) combined with muscle strength training in healthy young adults. All participants provided informed consent and received compensation for their time and effort.

### Participants

Healthy young adults were recruited through advertisements in the greater St. Louis community. Inclusion criteria were age 18 to 40 years and intact cognitive-motor functions to actively participate in the study. Exclusion criteria were: (1) a history of any neurological condition (i.e. stroke, i.e. stroke, Alzheimer's disease, Parkinson's disease), attention deficit disorder, attention deficit hyperactivity disorder, depression, bipolar disorder, balance impairment, or vestibular disorder; (2) history of depression or bipolar disorder; (3) recent wrist, hand or forearm injury that would affect their ability to lift weights; (4) any extremity, soft tissue, orthopedic, or vascular injury (i.e. peripheral vascular disease) which may contraindicate RLIC; (5) a history of sleep apnea which could confound the effects of RLIC [51, 52]; (6) any cognitive, sensory, or communication problem that might prevent completion the study; (7) current intensive weight lifting or interval training exercise, which could confound the effects of RLIC [53, 54]; (8) current substance abuse or dependence; (9) current use of medications, such as selective serotonin reuptake inhibitors, that could decrease nervous system excitability [55]; (10) participation in previous RLIC studies; and (11) an inability or unwillingness to travel for all study visits. As per the study protocol, the inclusion criteria of visual acuity of 20/50 with corrected vision and exclusion criteria of moderate to severe motion sickness were specific to cognitive testing (simulated driving task). Since this study reports only strength related measures, we stated inclusion and exclusion criteria specific to strength testing and training.

**Fig 1:**
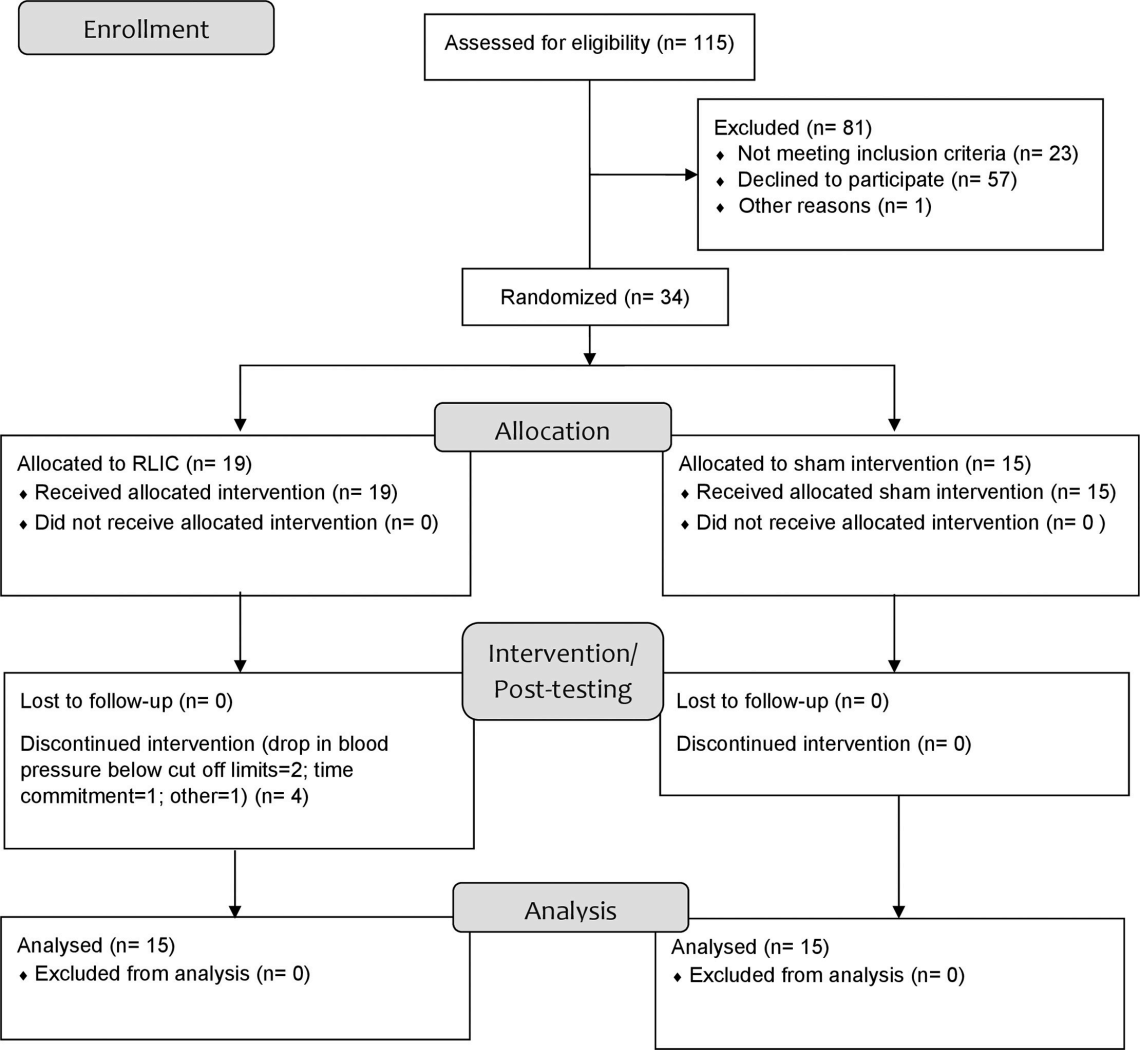
CONSORT flow diagram.

Fig 1 shows the CONSORT diagram, describing the flow of participants through the study. A total of 115 participants were assessed for eligibility and 34 participants were eventually randomized. Nineteen participants were assigned to receive RLIC and 15 to receive sham conditioning. Per the CONSORT diagram, 30 participants were included in the final analysis (RLIC: n = 15; sham: n = 15).

Data were collected in the Neurorehabilitation Research Lab of Washington University School of Medicine in St. Louis. Sample size was estimated based on previous studies of the effects of RLIC on a motor leaning task [56, 57]. Based on motor learning (change in balance score), 20 participants in each group was estimated to provide 80% power to detect a mean difference of 3 seconds change (posttest-pretest) between two treatment groups (RLIC vs. Sham) based on a two-sample t-test (significance level of 0.05). The standard deviations for change scores are assumed to be 2.5 seconds and 1.9 seconds for the RLIC and sham groups, respectively. Since we did not have pilot data for the effects of RLIC on strength, we performed post-hoc analysis to detect study power for strength measure. 15 participants in each group provided 82.6% power based on the strength measure.

### Experimental procedure

**Fig 2:**
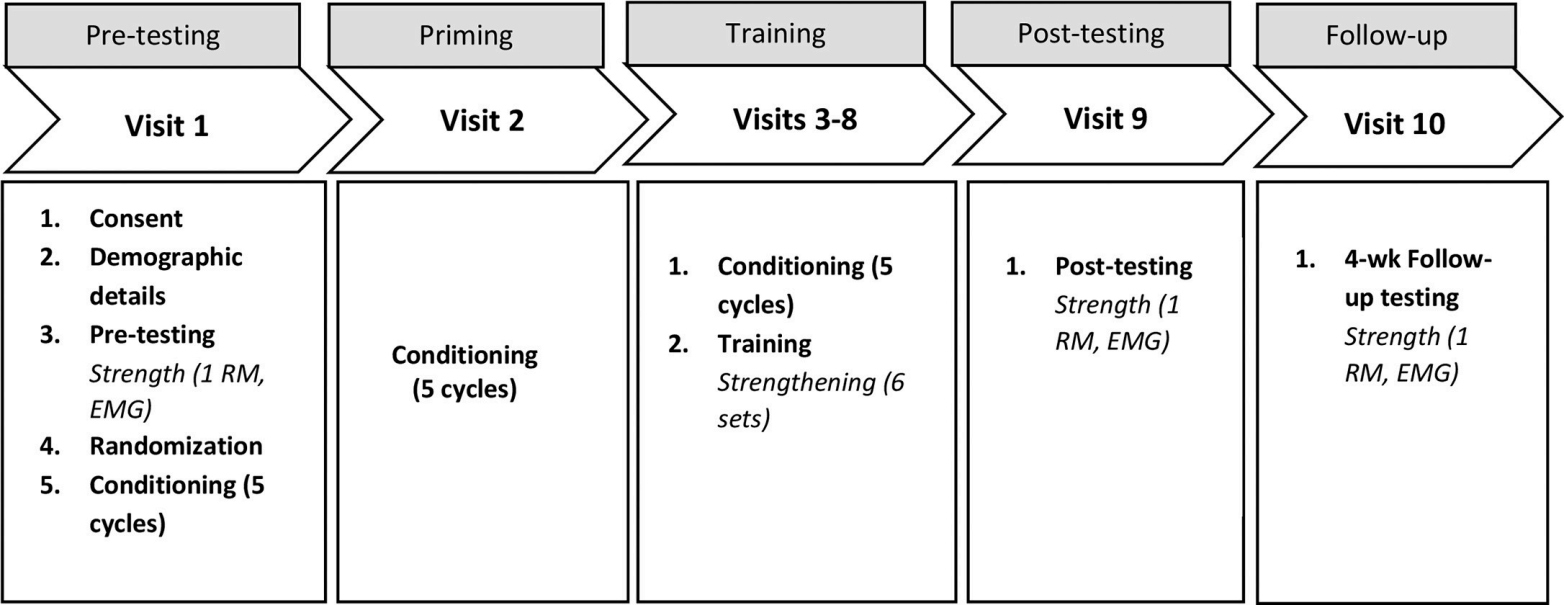
Order of experimental procedures.

The experimental procedure is shown in Fig 2. There were 10 total visits. Visits 1–3 occurred on consecutive weekdays. Visits 4–8 occurred on alternating weekdays to allow sufficient time between strengthening visits [58, 59]. Visit 9 occurred 1 weekday after visit 8, and visit 10, was a 4-week follow-up. During the first visit, participants provided demographic details including height and weight for the calculation of body mass index (BMI), and health and exercise history. After pre-testing, participants were randomly assigned to the RLIC or the sham group using a randomization list generated by the study statistician. Participants were blinded to group assignment until the completion of all 10 study visits.

#### Intervention

*Remote limb ischemic and sham conditioning*. Before beginning conditioning, we recorded resting heart rate, blood pressure, and oxygen saturation. Conditioning was performed by cyclic inflation and deflation of a blood pressure cuff on the dominant upper extremity. We specifically chose to perform conditioning on the dominant upper extremity (not the limb undergoing strength training), because the RLIC has systemic effects [60, 61]. Conditioning for the RLIC and sham conditioning groups was delivered as 5 cycles of 5 min blood pressure cuff inflation followed by alternating 5 minutes of deflation resulting in total conditioning time of 45 minutes [6, 25, 56, 62]. Consistent with our previous studies, the conditioning pressure in the RLIC group was ≥ 20 mmHg above that visit’s resting systolic blood pressure since this pressure was sufficient to induce ischemia and shown as effective as standard 200 mmHg, with fewer side effects [56, 57]. The conditioning pressure in the sham group was 10 mmHg below that visit’s diastolic BP since it gives the sensation of cuff inflation, but does not induce arterial occlusion [63–65]. All the participants received conditioning for the first 8 visits (Fig 1).

The experimenter continuously monitored the presence or absence of ischemia in the RLIC and sham conditioning groups, respectively, by monitoring a pulse oximeter placed on the index finger of the conditioning arm and by visually inspecting the color of the conditioning arm. A reading of “0” for pulse and oxygen saturation on the pulse oximeter and the presence of pale dusky appearance of the conditioning arm confirmed the presence of ischemia in the RLIC group. Oxygen saturation and pulse equivalent to baseline or prior to initiation of conditioning and unchanged color of the conditioning limb confirmed the absence of ischemia in the sham conditioning group. In the RLIC group, if the pulse or oxygen saturation reading appeared on the pulse oximeter anytime during the inflation cycle, the assessor increased the inflation pressure until confirmation of ischemia on the conditioning arm and the total time was adjusted to be consistent with 5 minutes of inflation cycle. Similarly, in the sham conditioning group, if oxygen saturation and pulse dropped below baseline measures, the conditioning pressure was decreased until preconditioning pulse and oxygen saturation was achieved and the arm showed no visible evidence of ischemia.

Conditioning safety was monitored through measurement of blood pressure, heart rate, oxygen saturation and pain on the non-conditioning arm before, during, and after each session. For both groups, conditioning was terminated if systolic BP was <85 or >160 mmHg; diastolic BP was <40 or >100 mmHg; heart rate was <60 or >100; oxygen saturation was <90%; or pain was >6 on Likert pain scale.

#### Strength training

We chose short duration (2 weeks), high intensity (80% of 1 RM) strength training in healthy young adults. The aim was to probe the effects of RLIC on muscle strength and early neural adaptation to short-term strength training, rather than to comprehensively evaluate strength, which would require a long-duration strength training programs [66–70]. Participants performed strength training within 5–25 minutes of conditioning. Therefore, strength training was performed within the early phase of the RLIC response [71].

A single wall-mounted pulley with stackable weights was used for strength training. The dynamic contraction strength of the wrist extensor muscles on the non-dominant extremity was trained. For strength training, participants were seated in a chair with trunk upright, feet firmly positioned on the floor, shoulder abducted to 45°, forearm pronated and strapped to a cushioned arm tray mounted on a table to avoid compensatory movements, wrist was in 80°- 85° flexion and participant firmly grasped the handle of a pulley, consistent with the previous study set up from our lab [72]. A total of 6 strength training sessions (visits 3–8) occurred on alternate weekdays across 2 weeks. Prior to each strength training session, participants performed warm-up for 2–3 minutes that included gentle wrist oscillations, stretching of wrist extensors, isometric contractions of wrist extensors, and wrist movements. The high intensity, progressive strength training protocol followed standard American College of Sports Medicine guidelines for intensity, frequency, duration, and progression [73]. Specifically, the participants completed 6 sets of wrist extension movement with 6–8 repetitions in each set. Resistance was set at 80% of the participant’s 1 Repetition Maximum (1 RM). Participants were verbally cued to slowly move the wrist, resulting in the concentric and eccentric phases of wrist movement for 5–6 seconds each. If the participant was unable to perform 6 repetitions per set with 80% of 1 RM intensity, the load was slightly reduced to ensure adequate stimulus for maximizing strength gains. Training was progressed over 6 visits by increasing resistance in subsequent visits when 8 repetitions were achieved in 4 out of 6 sets. Between each set, 1–3 minutes rest was provided since such inter-set rest intervals reduce metabolic stress and enhance strength gains [74]. Training load was calculated as the total repetitions in 6 sets during that visit multiplied by average weight across 6 sets.

#### Strength outcomes

*a*. *One Repetition Maximum (1 RM)*. Wrist extensor muscle strength of the non-dominant extremity was measured using 1 RM with the single column wall-mounted pulley with stackable weights. 1 RM was quantified as the maximum weight that a participant could lift only once from a position of full wrist flexion to full extension. Prior to the testing, participants were familiarized with the testing protocol and performed warm-up exercises as described previously in strength training.

The set-up for 1 RM testing and the participants’ positioning was the same as described above. Prior to each attempt, participants were instructed to contract with full effort, and move through the full active range from flexion to extension. Standardized verbal encouragement was given to enhance efforts and was consistent across participants. The measurement of 1 RM was considered valid and complete if the participant lifted the weight through full range of motion and then lowered the weight back to the starting position. One to three minutes of rest was allocated between each attempt to minimize fatigue and maximize performance [75, 76]. Consistent with the literature, strength changes were also expressed and analyzed as percent change [58].

*b*. *Electromyography (EMG) recording and analysis*. To assess neural adaptation to strength training, we recorded wrist extensor muscle activation using EMG. Raw EMG was obtained using a 2-channel EMG system (Noraxon Inc, USA) with the sampling frequency set at 1000 Hz. The target for the EMG signal activity was the extensor carpi radialis longus (ECRL) muscle on the non-dominant extremity during maximum voluntary isometric contraction (MVIC). Because of the close proximity of the wrist muscles, the recorded signals likely include some activity from adjacent wrist extensor muscles. Before placing the surface electrodes, skin was cleaned with an alcohol swab for the removal of dead cells and oil, thereby reducing skin impedance. Bipolar Ag-AgCl, disc shape surface electrodes with 5 mm diameter and 20 mm interelectrode distance were placed at the muscle belly in the direction of muscle fibers, according to surface EMG for non-invasive assessment of muscles (SENIAM) guidelines (http://www.seniam.org). The ground electrode was placed on the lateral epicondyle of the elbow. To ensure reliability of positioning of electrodes across pre-, post-, and follow-up testing, placement of electrodes was measured and recorded between two reference points: 1) distance between medial and lateral epicondyles and 2) intersection point between vertical distance from the first electrode to medial distance from lateral epicondyle.

EMG signals were recorded during a MVIC, using a similar position as the 1 RM testing, with the wrist in neutral position. Participants were instructed to produce maximum isometric wrist extension force within 30° wrist extension range by pushing against a handheld dynamometer placed on the dorsum of the wrist for 3 maximal effort contractions lasting 5 seconds each. One to three minutes rest between each contraction was given to prevent muscle fatigue [75, 76]. EMG recording was performed for each participant at the pre-, post, and follow-up testing.

EMG data were processed off-line in MATLAB R2016a (MathWorks, Natick, MA) with custom-written software. Signals were full-wave rectified and smoothed with a second-order Butterworth-filter using cutoff frequencies of 20 and 250 Hz for lower and upper band-pass, respectively [77]. EMG data were quantified by averaging the amplitude of the EMG activation during the middle 3000 ms for each trial. The three trials were then averaged to yield a single value representing ECRL activation for a given assessment point. EMG activation from pre- and post-testing was also expressed and analyzed as % change.

The assessor that performed conditioning also did strength training and testing. Post-test strength assessments were performed within 24–36 hours after the conditioning i.e. within the delayed phase of the RLIC response [71].

### Statistical analysis

Data were managed and stored in a secure REDCap database (Vanderbilt University, Nashville, TN) [78] and statistical analyses were performed in SPSS Statistics 24 (Version 24.0, IBM Corporation, Armonk, New York) with two-sided tests at a significance level of 0.05. Descriptive statistics for continuous variables (mean, standard deviation, median and range) and for categorical variables (frequency tables) were obtained for the study sample. Normality of the data was assessed using normal distribution plots. Since the data were normally distributed, independent t-test tests were used for the continuous variables of age, weight, height, BMI, pain, conditioning pressure, oxygen saturation, systolic and diastolic BP. Fisher Exact Tests were used for categorical variables such as gender, dominance, and race. A mixed model analysis of variance (ANOVA) with groups (RLIC and sham) as between-subject, and time (pre-, post-, and follow-up) as within-subject, factors was used to determine if there was a significant difference in 1 RM and EMG amplitude. A separate mixed model ANOVA with groups (RLIC and sham) as between-subject, and time (visits 3–8) as within-subject, factors was used to determine if there was a significant difference in training load. We were specifically interested in the group by time interaction effects. Significant interaction effects were followed by a Bonferroni post-hoc analysis. Independent t-tests were used to determine if significant differences existed between groups in percent change for 1 RM and EMG amplitude. P values equal to or less than 0.05 alpha were considered significant. Results in the text and graphs are presented as mean ± standard error. Post-hoc power analyses were performed using G*Power [79].

## Results

Table 1 shows baseline characteristics of the participants included in the study. There were no differences (all P values > 0.05) in the baseline characteristics between the RLIC and sham groups. There were no adverse events in either group, but two participants were withdrawn from the RLIC group. The baseline BP for these two participants was 85/65 and 91/6 mmHg. At the end of the conditioning, BP dropped to 83/62 and 77/49 for the participant 1 and 2 respectively. These BP were below our cuff-off threshold of systolic BP (<85 mmHg) and diastolic BP (<40 mmHg). Therefore, participants were withdrawn from the study. However, both the participants did not have any signs of distress. We followed-up with both the participants twice within 24 hours, who did not report any unusual symptoms.

**Table 1:** Demographic data.

Participants			
Characteristics	RLIC (n = 15)	Sham (n = 15)	Main effect of group (p)
Age (years)	25.5 ± 0.99	27.3 ± 1.11	0.225
Female/Male	10/5	10/5	1.00
Dominant side (R/L)	14/1	15/0	1.00
Weight (lbs)	140.4 ± 8.26	151.26 ± 6.86	0.321
Height (inches)	65.8 ± 1.01	65.93 ± 1.21	0.933
BMI (kg/ (m*m))	22.55 ± 0.79	24.56 ± 1.00	0.125
Resting Systolic BP (mmHg)	111 ± 3.09	117 ± 2.23	0.139
Resting Diastolic BP (mmHg)	71 ± 1.77	75 ± 1.98	0.239
Race			0.931
Caucasian	8 (53.3%)	8 (53.3%)	
African American	1 (6.7%)	2 (13.3%)	
Asian	5 (33.3%)	4 (26.7%)	
Other	1 (6.7%)	1 (6.7%)	

Values are numbers or mean ± SE. RLIC = remote limb ischemic conditioning; BMI = body mass index.

For the remaining subjects, conditioning was delivered as planned. Average cuff inflation pressure for the RLIC group (143 ± 3 mmHg) was, as expected, significantly higher than the sham conditioning group (65 ± 2 mmHg; P = 0.001). Ischemia occurred during conditioning cycles in the RLIC group. Oxygen saturation during conditioning on the conditioning arm was 0 ± 0% in the RLIC group and 97.93 ± 0.11% in the sham group (P = 0.001). Pain reports from the RLIC group (2.43 ± 0.20) were higher than those in the sham group (1.69 ± 0.23, P = 0.001). Individual ratings of pain were variable; no participant reported pain higher than 4/10. Average systolic and diastolic BP data for both groups across 8 visits is shown in S1 Appendix.

### Strength

**Fig 3:**
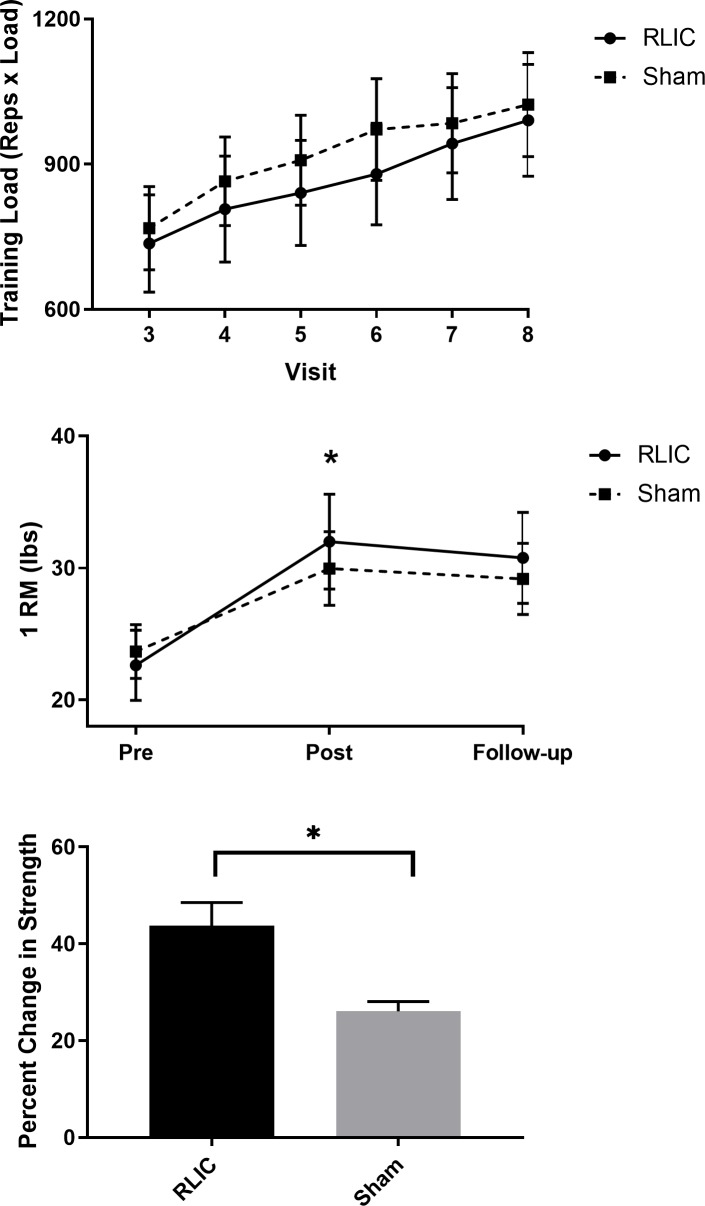
Strength. (A) Training load for each group during 2 weeks of strength training program. Training load was calculated as total number of repetitions by average load during each visit. Training visits 3–8 occurred on alternate weekdays. (B) 1 Repetition Maximum (1 RM) of the wrist extensor muscles on the non-dominant arm for each group. From pre- to post-test, mean change score in 1 RM in the RLIC group was 3.07 ± 0.75 lbs greater than the sham group. * indicates P<0.05 (group x time). (C) Percentage change in strength from pre- to post-test between groups. On average, the RLIC group demonstrated 17.67 ± 10.75% relative increase in wrist extensors strength compared to the sham group. * indicates P<0.05.

Overall, RLIC paired with strength training resulted in greater strength gains than sham conditioning paired with strength training. Average training load in the RLIC and sham groups during the 2 weeks is shown in Fig 3A. Both groups were trained at a similar workload over the 6 training sessions (visits 3–8) (P = 0.99).

Fig 3B shows 1 RM data at pre-, post-, and follow-up testing for each group. Both groups gained strength over time (main effect of time, P = 0.001). The RLIC group had a greater gain in strength compared to the sham group (group x time interaction, P = 0.035). No significant differences between groups were found overall (main effect of group, P = 0.844), or at specific time points (pre-, post-, and follow-up; P = >0.005). The greater strength gain was verified by a greater percent change in strength in the RLIC vs. sham group (P = 0.002, Fig 3C).

**Fig 4:**
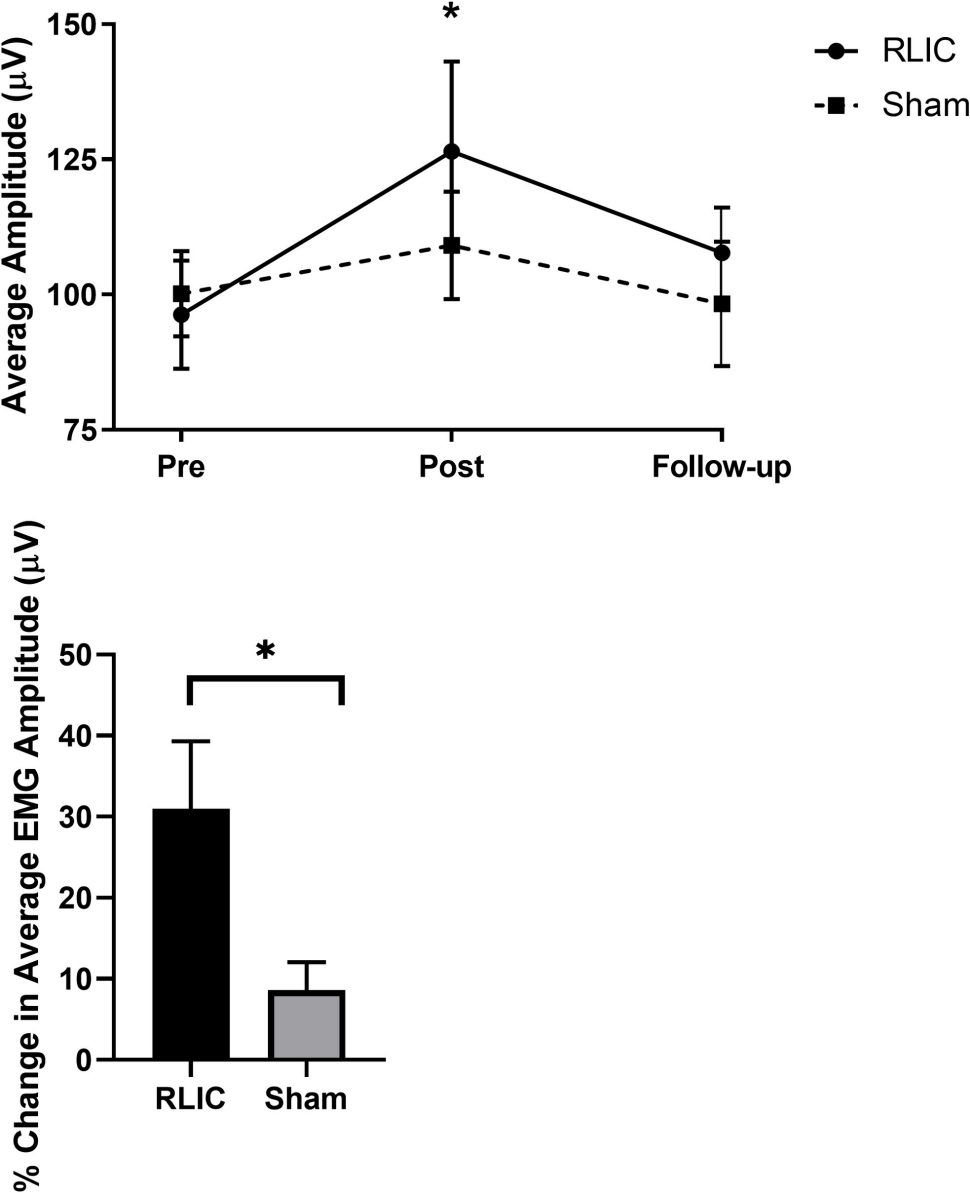
Electromyography (EMG). (A) EMG activity in extensor carpi radialis longus muscle on the non-dominant arm. Average amplitude of the EMG signal from pre- to post-test was increased in the RLIC compared to the sham group. (B) Percent change in EMG amplitude between from pre- to post-test between groups. On average, the RLIC group demonstrated 22.36 ± 4.89% relative increase in EMG amplitude compared to the sham group. Values are means ± SE. * indicates P<0.05.

The RLIC group also exhibited a trend towards greater neural adaptations to strength training as seen in Fig 4A. Both groups showed increased neural drive over time (main effect of time, P = 0.006). No significant differences in EMG amplitude were found between groups (main effect of group, P = 0.590), nor group by time interaction (P = 0.231). The RLIC group had greater % change in EMG amplitude than the sham group (P = 0.023, Fig 4B). Altogether, these results indicate that, as an adjunct to training, RLIC enhances strength gain.

Our post-hoc power analysis showed that with average 1 RM (Fig 3B), a standard deviation of 11 and a correlation of 0.97 among repeated measures of 1 RM from the observed data, a total sample of 30 (15 in each group) provided 82.6% power to detect a significant group x time interaction using two-sided tests based on mixed model analysis of repeated measures at alpha level of 0.05. With the observed average EMG amplitude (Fig 4A), a large standard deviation of 43 and a correlation of 0.69 among repeated measures, a total sample of 30 (15 in each group) provided only 29% power to detect a significant group x time interaction using two-sided F-tests based on mixed model analysis of repeated measures at significance level 0.05. Thus, there was sufficient statistical power to detect interactions for the strength measure, but not the neural adaptation measure.

## Discussion

This study investigated the effects of RLIC combined with strength training on strength gains in healthy young adults. The major finding of the present study is that combining RLIC with strength training increased wrist extensor muscle strength by 44%. The strength gains also appeared to be parallel with increases in muscle activation, potentially due to greater neural adaptations to strength training when combined with RLIC. Altogether these preliminary results suggest that RLIC holds promise to enhance skeletal muscle strength training.

To the best of our knowledge, this study represents the first time that RLIC paired with high intensity strength training has been tested and shown to facilitate muscle strength gain. This study applied RLIC to the dominant arm prior to strength training, and strength training and testing were performed on the non-dominant, non-conditioned arm. Thus, systemic effects of RLIC improved muscle strength, not local effects [22, 48]. Results of our study open the possibility that RLIC has the potential to enhance strength gains in a short period of time in individuals undergoing rehabilitation for various conditions that result in muscle weakness as well as to improve muscle performance in athletes. If the promise of this combinatorial therapy were to hold true in future, larger, later phase studies, then this clinically feasible, inexpensive paradigm could be easily implemented in clinics and exercise facilities.

The 44% strength gain seen in the RLIC group is notable with a 2-week strengthening duration. Strength training with longer durations (6–12 weeks) and similar intensities (> 70% of 1 RM) reported an average of 35% strength gains [80]. Two studies that used strength training protocols with short durations, and similar intensity, frequency, and progression showed 10% and 17% gains in ankle dorsiflexors over 2 weeks [58, 81], and 33% gains in hand muscles over 4 weeks [82]. Rapid, large improvements in strength could be highly relevant for many clinical populations. Our results from RLIC plus strength training are in line with previous reports showing the beneficial effects of a single session of RLIC on muscle performance in the absence of training [17, 18, 23, 25, 33]. It should be noted that our results showed significant group x time interaction in absolute strength gain; however, there were no significant group differences. Although RLIC group had a 3.03 lbs greater gain in strength compared to sham group from pre- to post-testing, we speculate that 2 weeks of strength training might not be sufficient for detecting group differences at post-intervention.

Given the exploratory nature of this study, we can only speculate about the possible vascular and cellular mechanisms underlying strength gains. Previous studies show that RLIC causes vasodilation due to release of several humoral factors [83, 84], increasing blood flow to skeletal muscles [85]. Such increased vasodilation might improve delivery of oxygen and nutrients to the increasing metabolic demands of muscles during strength training [20], thereby improving the performance of muscles [30, 86]. Secondly, RLIC attenuates lactate production [18], which potentially lowers sensitivity to fatigue signals [23]. Third, RLIC enhances muscle efficiency in ATP usage via ATP sparing and by improving efficiency of excitation-contraction coupling [12, 15, 87]. All these mechanisms might have contributed to the enhanced strength gains we witnessed when RLIC was paired with strength training.

Strength gains are often partially a function of neural adaptation to training [88]. Although the strength gains here were accompanied by a > 30% change in neural drive, these changes in neural adaptation achieved statistical significance between groups only when considered as a percent of baseline values. It is possible that the lack of group difference seen in EMG amplitudes stem from insufficient duration of training [59, 89]. More likely however, is that the large standard deviations in the RLIC group reduced our ability to find differences. Indeed, the percent change in EMG in RLIC group is comparable to changes elicited by 3–4 weeks of similar training, reflecting increased neural drive [59, 88–91]. Although the EMG results are mixed, the data are consistent with the findings from single-session studies that RLIC augments neural drive [25, 33, 92]. Others have shown that group III and IV afferent neurons in skeletal muscle are blocked by humoral factors released by RLIC [93, 94], which increases central motor drive and facilitates efferent neural drive to increase motoneuron activation [95, 96]. This is one potential underlying mechanism that might have facilitated greater neural adaptation to strength training when combined with RLIC.

The cardioprotection literature suggests short/early (0–12 hours) and long/delayed (24–72 hours) phase protection response to ischemic conditioning, which is mediated by different mechanisms [71]. In this study, we performed strength training within 5–25 minutes after the RLIC, i.e. within the early phase of RLIC response. The post-testing of strength was performed within 24–36 hours after the RLIC session, i.e. within the delayed phase of RLIC response. Although the mechanisms of the early and delayed phase of RLIC response to improve muscle performance are unknown, we speculate that RLIC mediated changes in the strength measures might differ between the early and delayed phases and should be investigated in the future studies. Moreover, it would be interesting to explore the effects of strength training performed during delayed phase on the strength outcomes. Future trial designs should include strength training after 24 hours of the RLIC session.

We recognize four main study limitations and propose future study directions based on these preliminary results. First, this is an early phase trial that combined RLIC with a short-duration strength training protocol. Although the data are promising, future studies combining RLIC with longer duration of strength training are warranted in healthy young and older populations. Biweekly assessments of strength during such studies are recommended in order to assess the time course over which benefits accrue. A second limitation is that the conditioning pressure used in the sham group was 10 mmHg below diastolic BP. Although this pressure did not cause ischemia in the conditioned arm, the average pressure in the sham group was comparable to pressure used in a blood flow restriction study [49]. The average 23% strength gains in the sham group, which was higher than expected [58, 81], could be a result of venous blood flow restriction on the conditioned arm affecting the training of the unconditioned arm. Thus the sham conditioning used here may not be a true sham group. A third limitation is that RLIC alone has been shown to increase strength and muscle performance, without any training [15, 17–22, 26], and we did not include such a group. Future study designs could include four groups (RLIC alone, RLIC + training, BFR + training, and a true sham + training) to compare the effects of all four protocols. Lastly, although we used standardized verbal encouragement for strength testing and training for all the participants, lack of accessor blinding could introduce potential bias in the strength measures. Future studies should incorporate double-blind design where both the participants and assessors are blinded to the intervention group assignment.

## Conclusions

When paired with strength training, RLIC facilitates greater strength gains in healthy young adults. RLIC is a safe, inexpensive, and clinically feasible method. Future randomized controlled trials and mechanistic studies are warranted in various populations that could benefit from this therapy. Testing this intervention in different populations such as older adults, and individuals with neurological injuries and/or chronic disease will help in understanding if the responses to RLIC vary among different populations, and whether this adjunct intervention can help these patients.

## Supporting information

S1 Checklist CONSORT 2010 checklist of information to include when reporting a randomised trial*. (DOC)

S1 Appendix (DOCX)

S1 Data (XLS)

S1 Protocol (RTF)
