# Supplementary material for: Effects of remote limb ischemic conditioning on muscle strength in healthy young adults: A randomized controlled trial
Source: PLoS One. 2020 Feb 4;15(2):e0227263. doi: 10.1371/journal.pone.0227263 (PMC6999897; doi:10.1371/journal.pone.0227263)
Supplement: S1 Appendix — (DOCX) [file pone.0227263.s002.docx]

Table 2: Average changes in systolic and diastolic blood pressure (mmHg) in the RLIC and sham group across 8 visits.

| **RLIC Systolic BP (mmHg)** | | | |
| --- | --- | --- | --- |
| Visits | Pre-conditioning | During-conditioning | Post-conditioning |
| 1 | 112 | 110 | 111 |
| 2 | 113 | 107 | 107 |
| 3 | 112 | 106 | 107 |
| 4 | 109 | 106 | 106 |
| 5 | 109 | 109 | 105 |
| 6 | 110 | 105 | 104 |
| 7 | 111 | 108 | 105 |
| 8 | 112 | 106 | 108 |

| **RLIC Diastolic BP (mmHg)** | | | |
| --- | --- | --- | --- |
| Visits | Pre-conditioning | During-conditioning | Post-conditioning |
| 1 | 71 | 72 | 70 |
| 2 | 71 | 69 | 66 |
| 3 | 70 | 69 | 63 |
| 4 | 67 | 67 | 63 |
| 5 | 76 | 69 | 64 |
| 6 | 72 | 71 | 64 |
| 7 | 77 | 70 | 65 |
| 8 | 70 | 70 | 65 |

| **Sham Systolic BP (mmHg)** | | | |
| --- | --- | --- | --- |
| Visits | Pre-conditioning | During-conditioning | Post-conditioning |
| 1 | 117 | 116 | 115 |
| 2 | 117 | 114 | 116 |
| 3 | 117 | 112 | 114 |
| 4 | 118 | 113 | 111 |
| 5 | 115 | 111 | 109 |
| 6 | 117 | 108 | 110 |
| 7 | 119 | 113 | 114 |
| 8 | 116 | 113 | 109 |

| **Sham Diastolic BP (mmHg)** | | | |
| --- | --- | --- | --- |
| Visits | Pre-conditioning | During-conditioning | Post-conditioning |
| 1 | 75 | 76 | 75 |
| 2 | 76 | 75 | 76 |
| 3 | 76 | 73 | 73 |
| 4 | 74 | 74 | 72 |
| 5 | 75 | 73 | 73 |
| 6 | 75 | 73 | 73 |
| 7 | 75 | 72 | 71 |
| 8 | 73 | 73 | 72 |
