# Supplementary material for: Effects of remote limb ischemic conditioning on muscle strength in healthy young adults: A randomized controlled trial
Source: PLoS One. 2020 Feb 4;15(2):e0227263. doi: 10.1371/journal.pone.0227263 (PMC6999897; doi:10.1371/journal.pone.0227263)
Supplement: S1 Protocol — (RTF) [file pone.0227263.s004.rtf]

Remote Limb Ischemic Conditioning to Enhance Performance, Learning And Muscle Strength
	
Study Objectives	The objective of this research study is to determine if remote limb ischemic conditioning (RLIC) can enhance learning of an ecologically valid, complex cognitive-motor (driving) task and increase skeletal muscle strength in neurologically intact young adults.	
Background & Rationale	Ischemic conditioning is an endogenous phenomenon in which exposing a target organ or tissue to one or more brief episodes of ischemia results in protection of that organ against subsequent ischemia.1 The effects of ischemic conditioning are not confined within an organ but can be can be transferred from one organ to another, a technique called remote ischemic conditioning.2 A clinically feasible method for this is remote limb ischemic conditioning (RLIC), where episodes of ischemia and perfusion are induced with a blood pressure cuff placed on the arm.3

In humans, the cardioprotective effects of RLIC have been demonstrated.4,5 For example, applying an inflated blood pressure cuff to the upper or lower limb has shown efficacy for protection in people undergoing cardiac surgeries,4,6 elective surgery to repair abdominal aortic aneurysm,7 experiencing myocardial infarction,5 and with symptomatic intracranial arterial stenosis.6 Although the cardioprotective effects of RLIC are well established, the neuroprotective effects are just beginning to be evaluated.6 Moreover, the effects of RLIC on skeletal muscle is largely unknown. Only a single study so far has shown that RLIC decreases depletion of Adenosine triphosphate (ATPs), decreases lactate accumulation and increases myeloperoxidase, which helps in reducing inflammation and apoptosis (cell death) in skeletal muscles of rats.8

This research study is a part of larger phase I series of experiments that is investigating if the effects of RLIC might extend beyond cardio- and neuroprotection and into plasticity, learning and recovery. The preliminary results of the proof-of-concept study from our lab has shown that RLIC robustly facilitates motor learning and retention in young, neurologically intact adults.9 However, these learning effects have only been seen in a highly controlled balance task. Hence, the next step is to investigate if RLIC can enhance performance of an ecologically valid complex cognitive-motor task such as driving in a simulated environment and increase skeletal muscle strength in healthy young adults. 

This study is important because if eventually effective, RLIC could have profound effect on the rehabilitation and recovery of cognitive-motor function and muscle strength in people with neurological injury, such as stroke. 	
Study Design	Between groups repeated measures design. 
	
Groups	Group 1: Neurologically intact subjects receiving RLIC
Group 2: Neurologically intact subjects receiving sham conditioning

Subjects who qualify and consent will be randomly assigned to either group 1 or 2.  Participants will be blinded to the group assignment..	
Number of Subjects & Power Analysis	Based on our pilot study9 and an earlier study on strength training10,
we need 20 participants in each group (total 40). To account for withdrawals and participants lost to follow-up, we request permission to enroll 60 subjects.

For the balance task, this will provide at least 80% power to detect a mean difference of 3 seconds change (posttest - pretest) between two treatment groups (RLIC vs. Sham) based on a two-sample t-test (significance level of 0.05). The standard deviations for change scores are assumed to be 2.5 seconds and 1.9 seconds for the RLIC and sham groups, respectively. For the strength task, this give 98% power to detect mean difference of 17 % change in the muscle strength (posttest – pretest) after 2-weeks strength training based on a two-sample t-test at a significance level of 0.05. The standard deviations for change scores are assumed to be 4.90 kg and 5.53 kg for pre- and post-strength training respectively. 
	
Inclusion & Exclusion Criteria	Inclusion Criteria: 
1) Healthy adults between the age of 18 and 40 years 
2) Visual acuity of 20/50 with corrected vision, which is the required visual acuity as per the state guidelines for driving 

Exclusion Criteria: 
1) History of neurological condition (i.e. stroke, Alzheimer's disease, Parkinson's disease), ADD, ADHD, depression, bipolar disorder, balance impairment, or vestibular disorder 
2) History of severe motion sickness, moderate to severe motion sickness or nausea on oculo-motor components of Simulator sickness questionnaire (Appendix 1)11, inability to ride a car, boat, train or airplane due to motion sickness
3) Recent wrist, hand or forearm injury that would currently prevent ability to lift weights
4) History of lower extremity condition, injury, or surgery that would currently impair ability to stand or balance 
5) Any extremity soft tissue, orthopedic, or vascular injury (i.e. peripheral vascular disease) which may contraindicate RLIC 
6) Any cognitive, sensory, or communication problem that would prevent completion of the study 
7) History of or current sleep apnea
8) Current intensive weight lifting or interval training exercise 
9) Current substance abuse or dependence 
10) Unwillingness to travel for all study visits 
	
RLIC and Sham Conditioning
	Remote limb ischemic conditioning will be achieved via blood pressure cuff inflation to 20 mmHg above systolic blood pressure on the dominant, upper extremity.12 Sham conditioning will be achieved via blood pressure cuff inflation to 10 mmHg under diastolic blood pressure on the dominant, upper extremity. Conditioning will involve 5 cycles of 5 minutes blood pressure cuff inflation followed by alternating 5 minutes of cuff deflation. Subjects will be blinded to their group assignment (RLIC or sham conditioning). Conditioning requires 45 minutes. RLIC or sham conditioning will be performed on visits 1-8.	
Behavioral Training 	Three tasks will be used for behavioral training on visits 3-8. 

1)	Driving task (15-20 minutes)
2)	Strengthening task (20-30 minutes)
3)	Balance training task (15 minutes; rest breaks as needed)

Driving task: The driving task requires the participant to drive in a simulated environment of a driving simulator (STISIM M300WS, Systems Technology, Inc.). The simulator presents a realistic driving experience through controlled, complex tasks related to vehicle dynamics, natural pedal and steering wheel controls, and a wide field of view display. For the driving task, participants will drive through increasingly complex scenarios (complex urban, suburban, rural, and construction zone). Hazards, maneuvers and basic rules of the road are presented to the participant during the scenarios. Performance will be quantified on a weighted composite score for time to scenario completion, hitting a pedestrian, collisions, center lane crossing, off road accidents, stop sign and traffic light rules, and other driving errors. 

Strengthening task: Strengthening of wrist extensor muscles on the non-dominant extremity was chosen because wrist extensor weakness can be a main impairment limiting activities of daily living and participation and is often the focus of many upper extremity rehabilitation interventions.13 Participants will sit with shoulder and forearm supported so that motion is isolated to the wrist. A single column wall-mounted pulley with stackable weights will be used. Prior to training, the participant will be guided through a warm-up to maximize performance. Training will be initially set at 6 sets of 6-8 repetitions at 80% of 1 repetition maximum (RM), which would be the required high intensity to achieve training effects.14 Training will be progressed by increasing the weight over sets. We will provide up to 5 minutes of rest after each set to prevent muscle fatigue and soreness.

Balance Task: The balance task requires subjects to stand on a movable platform (Stability Platform, model 16030L, Lafayette Instrument) and to keep the platform in a balanced, horizontal position.15 This task was selected because it can be easily modified to the appropriate level of difficulty in accordance with each subject's motor abilities.
	
Descriptive measures	Demographic information, including age, dominant side, gender, ethnicity, race, level of physical activity, height, body weight, employment status, education level, co-morbidities, current medications and history of motion sickness will be collected on all subjects on visit 1. Performing all these descriptive measures on visit 1 will allow us to determine the inclusion/exclusion of the participant in the study.	
Outcome Measures	The outcomes measured include-
Performance on:
           1. Driving task
           2. Strengthening task
           3. Balance task.

Performance on the driving task will be measured by composite score, where the score=Time x Errors 
·	Time=time to completion of entire driving task
·	Errors= number of times: hitting a pedestrian, collisions, center lane crossing, off road accidents, stop sign and traffic light rule violations, and other driving errors. 

Performance on the strengthening task will be assessed by-
a.	One repetition maximum (1RM) of wrist extensors: Maximum amount of force (kilograms) that can be generated in one contraction.16 1 RM will be quantified as the maximum amount of weight (kg) that a participant can lift in one repetition that will be placed on a single column wall-mounted pulley. Participants will sit with shoulder and forearm supported on a table, with shoulder abducted to 450, elbow flexed to 900, and wrist flexed. 
b.	Maximum Voluntary Isometric Contraction (MVIC) of wrist extensors: Maximum amount of isometric force (kg) exerted by wrist extensor muscles against the hand held dynamometer.17 Dynamometry is a standardized method for measurement of muscle strength. For the strength testing, the participant will sit with forearm supported on a table, shoulder abducted to 450, elbow flexed to 900, and wrist slightly flexed. We will ask the participant to produce maximum wrist extension force by pushing against the dynamometer placed on the dorsum of the wrist for three maximal-effort contractions lasting 5 seconds each with 2 minutes rest between each contraction. As a standard strength measure with the dynamometer, the average of three contractions will be considered as MVIC for wrist extensor muscles.
c.	Electromyography (EMG): The EMG data will be used to quantify the electrical amplitude of Extensor Digitorum Communis (EDC) muscle. Increase in the amplitude of EMG is an indicator of neurophysiological adaptation to strength training.10,18 We will simultaneously record the EMG activity of EDC muscle while performing MVIC. EMG data will be gathered using 2-channel EMG system (Noraxon Inc, USA).The active electrodes will be placed on the belly of EDC. Two active electrodes will be separated with 2 centimeters distance and the ground electrode will be placed on the bony prominence (lateral epicondyle) of the elbow.

Performance on the standing balance task will be quantified by identifying the number of seconds in a 30-second trial that an individual is able to maintain the platform within ±3° of horizontal.
	
Order of Experiment/Study Visits	This study involves 10 total visits. Participants will be in the study for up to 7 weeks.  Please refer to Table 1 for a timeline of study visits.
Visit 1: We will provide informed consent and gather demographic data. Next we will perform baseline assessments for the performance on the driving task, the strengthening task and the balance task.
            For quantifying the performance on the driving task, subjects will perform 15-20 min driving task in the driving simulator. We will monitor the participants' symptoms for simulator sickness and administer the Simulator Sickness Questionnaire (SSQ) at the end of the driving task.
            Then, we will quantify muscle strength of the non-dominant wrist extensors by recording 1 RM, MVIC and EMG activity of wrist extensor muscles.
            Next, we will gather measurements on balance task. 
            Subjects will then be randomized to their treatment group. After randomization, subjects will undergo RLIC or sham conditioning (see RLIC and Sham Conditioning for the details). This visit will take approximately: 2.5 hours.

Visit 2: Subjects will undergo RLIC or sham conditioning This visit will take approximately: 1 hour.

Visit 3-8: Subjects will undergo RLIC or sham conditioning.  Next, behavioral training will commence (driving task, strengthening task, and balance training). The order of behavioral training task practice will be randomized. Visits 3-8 will occur every alternate business day. These visits will take approximately: 2 hours.

Visit 9: Subjects will complete post-testing which will include a) 15-20 min driving task; b) 1 RM and MVIC of the wrist extensor muscles on the non-dominant arm; c) EMG of the EDC muscle on the non-dominant arm, and d) balance testing. Visit 9 will be on the next business day after visit 8. During this visit, participants will not receive RLIC or sham conditioning. This visit will take approximately: 1 hour. 

Visit 10: Post-test performance assessment on driving test, 1 RM and MVIC and EMG of the EDC muscle on the non-dominant arm, and balance testing will be performed after 4-weeks from the visit 9. Participants will not receive RLIC or sham conditioning during the follow-up visits. Participants will fill out a survey on the follow-up visit indicating whether they think that they received RLIC or sham treatment. This visit will take approximately: 1 hour.
	
Data Analysis	Data will be analyzed using a mixed model ANOVA with time (pretest, posttest, and follow-up) as within subject factor and group (RLIC vs. sham treatment) as between subject factor. 
	
Safety Considerations & Monitoring	The risks of participating in the rehabilitation intervention are minimal and are similar to the risks encountered during routine physical and occupational therapy services. 

Conditioning: 
The likely risks are: 
       1. Bruising on the conditioning arm: To avoid bruising from the pressure cuff, participant's arm will be covered with a folded cotton pillowcase.
       2. Discomfort:  To avoid discomfort in the conditioning arm during each conditioning cycle, we will ask the participant about discomfort. If the participant reports discomfort, we will reposition the pressure cuff on the arm and allow the participant to move the arm during 5-minutes of deflation cycle.
      3. Pain or tingling on the conditioning arm: To avoid pain or tingling in the conditioning arm during the conditioning cycle, we will ask the participant about pain and tingling. If the participant reports pain or tingling, we will reposition the pressure cuff on the arm and allow the participant to move the arm during 5-minutes of deflation cycle.

Driving: 
The likely risks are
1.	Motion sickness that can result in dizziness, nausea, yawning, perspiration, excessive blinking, vertigo, headache and general discomfort: To avoid motion sickness, the temperature of the driving simulation room will be regulated; we will keep the room cool and turn the fan on to maintain ventilation. We will also closely visually monitor early signs of motion sickness. Moreover, if the participant reports motion sickness during the training, we will immediately stop the simulator and will ask the participant to relax in the simulator chair until the symptoms subside. However, if motion sickness persists, participant will not resume the simulated driving training on that day and we will ask the participant to monitor the symptoms until before the start of the next visit. If motion sickness persists until next visit, we will discontinue participation from driving training.
2.	 Anxiety while performing a simulated driving task: To reduce anxiety, we will reassure the participants that their performance is in a simulated driving environment and will not impact their ability to drive on road.

Strengthening: 
The likely risks are
1.	Wrist pain: To avoid wrist pain, participants will perform warm-up exercises for 5 minutes before starting the strengthening.
2.	Muscle fatigue and delayed onset muscle soreness: Strength training on every alternate day will allow sufficient time to recover from muscle fatigue and delayed onset muscle soreness. 


Balance: 
The less likely risks are
1. Falling from the balance board: To avoid falling from the balance board, participants will be permitted to use a handrail as needed for safety during balance task and up to two research team members will supervise the balance performance. Participants will also wear a gait belt for safety, which will help the research team member to catch the participant if they start to fall.


In order to monitor for further overall safety, we will continuously monitor lightheadedness, respiratory distress, cyanosis, and spasms. We will record and monitor heart rate, blood pressure, and oxygen saturation before, during, and after each session of RLIC. Sessions will be terminated if heart rate <40 bpm or >160bpm, systolic BP <85mmHg or >160mmHg, diastolic BP <40 or >100 mmHg or if O2 saturation <90%. Moreover, during the follow up session, all subjects will be asked to fill out a questionnaire asking about any adverse effects that resulted from participation in this study. 	
Investigators	PI: Catherine Lang PT, PhD
Study Coordinator: Swati Surkar, PhD, PT
	
Number of Centers	1 center: Washington University School of Medicine	
Key References	Please see the reference list.	


References
1.	Gidday, J. M. (2006). "Cerebral preconditioning and ischaemic tolerance." Nat Rev Neurosci 7(6): 437-448.
2.	Saxena, P., et al. (2010). "Remote ischemic conditioning: evolution of the concept, mechanisms, and clinical application." J Card Surg 25(1): 127-134.
3.	Kharbanda, R. K., et al. (2009). "Translation of remote ischaemic preconditioning into clinical practice." Lancet 374(9700): 1557-1565.
4.	Hausenloy, D. J., et al. (2007). "Effect of remote ischaemic preconditioning on myocardial injury in patients undergoing coronary artery bypass graft surgery: a randomised controlled trial." Lancet 370(9587): 575-579.
5.	Botker, H. E., et al. (2010). "Remote ischaemic conditioning before hospital admission, as a complement to angioplasty, and effect on myocardial salvage in patients with acute myocardial infarction: a randomised trial." Lancet 375(9716): 727-734.
6.	Meng, R., et al. (2012). "Upper limb ischemic preconditioning prevents recurrent stroke in intracranial arterial stenosis." Neurology 79(18): 1853-1861.
7.	Ali, Z. A., et al. (2007). "Remote ischemic preconditioning reduces myocardial and renal injury after elective abdominal aortic aneurysm repair - A randomized controlled trial." Circulation 116(11): I98-I105.
8.	Addison, P. D., et al. (2003). "Noninvasive remote ischemic preconditioning for global protection of skeletal muscle against infarction." American Journal of Physiology-Heart and Circulatory Physiology 285(4): H1435-H1443.
9.	Cherry-Allen, K. M., et al. (2015). "Remote limb ischemic conditioning enhances motor learning in healthy humans." J Neurophysiol 113(10): 3708-3719.
10.	Christie, A., & Kamen, G. (2010). “Short‐term training adaptations in maximal motor unit firing rates and afterhyperpolarization duration.” Muscle & nerve, 41(5), 651-660.
11.	Kennedy, R.S., Lane, N.E., Berbaum, K.S., & Lilienthal, M.G. (1993). “Simulator Sickness Questionnaire: An enhanced method for quantifying simulator sickness.” International Journal of Aviation Psychology, 3(3), 203-220

12.	Cherry-Allen, K. M., Gidday, J. M., Lee, J. M., Hershey, T., & Lang, C. E. (2017). “Remote Limb Ischemic Conditioning at Two Cuff Inflation Pressures Yields Learning Enhancements in Healthy Adults.” Journal of motor behavior, 49(3), 337-348.

13.	Hakkennes, S., & Keating, J. L. (2005). “Constraint-induced movement therapy following stroke: a systematic review of randomised controlled trials.” Australian Journal of Physiotherapy, 51(4), 221-231.


14.	Jenkins, N. D., Miramonti, A. A., Hill, E. C., Smith, C. M., Cochrane-Snyman, K. C., Housh, T. J., & Cramer, J. T. (2017). “Greater Neural Adaptations following High-vs. Low-Load Resistance Training.” Frontiers in physiology, 8.

15.	Taubert, M., Draganski, B., Anwander, A., Müller, K., Horstmann, A., Villringer, A., & Ragert, P. (2010). “Dynamic properties of human brain structure: learning-related changes in cortical areas and associated fiber connections.” Journal of Neuroscience, 30(35), 11670-11677.

16.	Shimano, T., Kraemer, W. J., Spiering, B. A., & Volek, J. S. (2006). “Relationship between the number of repetitions and selected percentages of one repetition maximum in free weight exercises in trained and untrained men.” Journal of Strength and Conditioning Research, 20(4), 819.


17.	Meldrum, D., Cahalane, E., Conroy, R., Fitzgerald, D., & Hardiman, O. (2007). “Maximum voluntary isometric contraction: reference values and clinical application.” Amyotrophic Lateral Sclerosis, 8(1), 47-55.

18.	Carroll, T. J., Selvanayagam, V. S., Riek, S., & Semmler, J. G. (2011). “Neural adaptations to strength training: moving beyond transcranial magnetic stimulation and reflex studies.” Acta physiologica, 202(2), 119-140.


Table 1: Order of the 10 study visits. Testing occurs on Study Visits 1, 9, and 10. Conditioning which involves 5 cycles of 5 minutes of upper extremity peripheral ischemia (or sham), alternating by 5 minutes of no ischemia, occurs on Study Visits 1-8. Training occurs on Study Visits 3-8 (Study Visits 4-8 occur every other business day, excluding weekends and holidays) and includes driving simulation, strengthening, and balance training.


Assessments	
Visits	
	1
(Business Day 1)	2
(Business Day 2)	 3
(Business Day 3)	4
(Business Day 5)	5
(Business Day 7)	6
(Business Day 9)	7
(Business Day 11)	8
(Business Day 13)	9
(Business Day 14)	10
(4 weeks from visit 9)
	
Consent/
Demographic details	✔										
Pre-testing 	✔
										
Conditioning 
(RLIC/
Sham)	✔	✔	✔	✔	✔	✔	✔	✔			
Training
			✔	✔	✔	✔	✔	✔			
Post-testing									✔	✔	


1.	Pre-testing includes: Performance on the- 1) Driving task, and 2) Strengthening task, and 3) Balance task.
2.	Training includes: Training on the- 1) Driving task with the driving simulator, 2) Wrist Extensor Strengthening (non-dominant arm), 3) Balancing task on the balance board.
3.	Post-testing includes: Performance on the- 1) Driving task, and 2) Strengthening task, and 3) Balance task.


Appendix 1

No.______________ 							Date____________________
SIMULATOR SICKNESS QUESTIONNAIRE
Kennedy, Lane, Berbaum, & Lilienthal (1993)***
Instructions : Circle how much each symptom below is affecting you right now. 
1. General discomfort				None 		Slight 		Moderate 	Severe 2. Fatigue					None 		Slight 		Moderate 	Severe 3. Headache					None 		Slight 		Moderate 	Severe 4. Eye strain 					None 		Slight 		Moderate	 Severe 5. Difficulty focusing 				None 		Slight 		Moderate 	Severe 6. Salivation increasing 				None 		Slight 		Moderate 	Severe 7. Sweating 					None 		Slight 		Moderate 	Severe 8. Nausea				 	None 		Slight 		Moderate 	Severe 9. Difficulty concentrating 			None 		Slight 		Moderate 	Severe 10. Fullness of the Head 			None 		Slight 		Moderate 	Severe 11. Blurred vision 				None 		Slight 		Moderate 	Severe 12. Dizziness with eyes open 			None 		Slight 		Moderate 	Severe 13. Dizziness with eyes closed 			None 		Slight 		Moderate 	Severe 14. *Vertigo					None 		Slight 		Moderate 	Severe 15. **Stomach awareness 			None 		Slight		Moderate 	Severe 16. Burping 					None 		Slight 		Moderate 	Severe 

Total: items 1 to 16 (scale of 0 to 3). 
 Nausea: items 1 + 6 + 7 + 8 + 12 + 13 + 14 + 15 + 16. 
Oculo-motor: items 2 + 3 + 4 + 5 + 9 + 10 + 11.

* Vertigo is experienced as loss of orientation with respect to vertical upright.
** Stomach awareness is usually used to indicate a feeling of discomfort which is just short of nausea. 
Last version : March 2013 ***Original version : Kennedy, R.S., Lane, N.E., Berbaum, K.S., & Lilienthal, M.G. (1993). Simulator Sickness Questionnaire: An enhanced method for quantifying simulator sickness. International Journal of Aviation Psychology, 3(3), 203-220
